# Bach1 Induces Endothelial Cell Apoptosis and Cell-Cycle Arrest through ROS Generation

**DOI:** 10.1155/2016/6234043

**Published:** 2016-02-29

**Authors:** Xinhong Wang, Junxu Liu, Li Jiang, Xiangxiang Wei, Cong Niu, Rui Wang, Jianyi Zhang, Dan Meng, Kang Yao

**Affiliations:** ^1^Department of Physiology and Pathophysiology, School of Basic Medical Sciences, Fudan University, Shanghai 200032, China; ^2^Laurentian University, Sudbury, ON, Canada P3E 2C6; ^3^Division of Cardiology, Department of Medicine, Stem Cell Institute, University of Minnesota Medical School, MN 55455, USA; ^4^Shanghai Institute of Cardiovascular Disease, Department of Cardiology, Zhongshan Hospital, Fudan University, Shanghai 200032, China

## Abstract

The transcription factor BTB and CNC homology 1 (Bach1) regulates genes involved in the oxidative stress response and cell-cycle progression. We have recently shown that Bach1 impairs cell proliferation and promotes apoptosis in cultured endothelial cells (ECs), but the underlying mechanisms are largely uncharacterized. Here we demonstrate that Bach1 upregulation impaired the blood flow recovery from hindlimb ischemia and this effect was accompanied both by increases in reactive oxygen species (ROS) and cleaved caspase 3 levels and by declines in the expression of cyclin D1 in the injured tissues. We found that Bach1 overexpression induced mitochondrial ROS production and caspase 3-dependent apoptosis and its depletion attenuated H_2_O_2_-induced apoptosis in cultured human microvascular endothelial cells (HMVECs). Bach1-induced apoptosis was largely abolished when the cells were cultured with N-acetyl-l-cysteine (NAC), a ROS scavenger. Exogenous expression of Bach1 inhibited the cell proliferation and the expression of cyclin D1, induced an S-phase arrest, and increased the expression of cyclin E2, which were partially blocked by NAC. Taken together, our results suggest that Bach1 suppresses cell proliferation and induces cell-cycle arrest and apoptosis by increasing mitochondrial ROS production, suggesting that Bach1 may be a promising treatment target for the treatment of vascular diseases.

## 1. Introduction

The transcription factor BTB and CNC homology 1 (Bach1) regulates genes involved in apoptosis, the oxidative stress response, mitotic chromatin dynamics, and the cell-cycle [1–5]. Previous studies suggest that Bach1 deficiency may protect against oxidative tissue damage in murine models of lung, liver, intestine, pancreas, and cardiovascular disease [6–11], and we have shown that Bach1 suppresses angiogenesis in mice with surgically induced hindlimb ischemia (HLI) [2]. Our results also indicate that Bach1 disrupts Wnt/*β*-catenin signaling and that this disruption reduces the proliferation, migration, and tube formation activity of endothelial cells (ECs) [12]. Bach1 upregulation also increased apoptosis in cultured ECs, but the mechanisms involved in Bach1-induced EC apoptosis are largely uncharacterized.

Physiological levels of reactive oxygen species (ROS), such as superoxide, hydrogen peroxide, and hydroxyl radical, are required for normal endothelial activity [13]; for example, we have shown that inhibition of NADPH oxidase subunit 4, which is among the most prominent sources of ROS production in the endothelium [14], impairs the migration, proliferation, and tube formation of human microvascular endothelial cells (HMVECs) [15]. However, the high ROS levels caused by inflammation and the response to ischemic injury appeared to impair neovascularization in the ischemic limbs of mice [16, 17] by inducing endothelial dysfunction and apoptosis [17]. The role of ROS in Bach1-induced apoptosis has not been investigated. Thus, in the present study, we conducted a series of analyses in a murine HLI model and in cultured HMVECs to determine whether the proapoptotic activity of Bach1 in ECs is mediated by ROS production.

## 2. Materials and Methods

### 2.1. Reagents

N-Acetyl-L-cysteine (A7250), H_2_O_2_ (88597), and the *β*-actin antibody (A5316) were purchased from Sigma-Aldrich (St. Louis, MO). Dihydroethidium (DHE, D1168) and MitoSOX Red (M36008) were obtained from Invitrogen (Carlsbad, CA). Antibodies against Bach1 (sc-14700), Bcl2 (sc-7382), Bcl-xL (sc-8392), HO-1 (sc-1796), cyclin D1 (sc-20044), cyclin E2 (sc-28351), cyclin B1 (sc-595), CDK2 (sc-163), CDK4 (sc-601), CDK6 (sc-177), p21 (sc-397), p53 (sc-126), the human Bach1 siRNA (sc-37064), and the control siRNA were obtained from Santa Cruz Biotechnology (Santa Cruz, CA). Antibodies against cleaved caspase 3 (9664) and cyclin A2 (4656) were obtained from Cell Signaling (Beverly, MA). The recombinant adenoviruses coding for human Bach1 (Ad-GFP-Bach1) or GFP (Ad-GFP) were purchased from GenePharma (Shanghai, China).

### 2.2. Cells

Human microvascular endothelial cells (HMVECs) were immortalized with the human telomerase catalytic protein (hTERT) [18] and obtained as a kind gift from Dr. Rong Shao (University of Massachusetts, Springfield, MA). The cells were cultured as described previously.

### 2.3. Mice

Eight-week-old male C57BL/6J mice were obtained from the Nanjing Biomedical Research Institute of Nanjing University (Nanjing, China) and housed in a standard, pathogen-free facility. All experimental procedures were approved by the Ethics Committee of Experimental Research at Fudan University Shanghai Medical College and were consistent with US National Institutes of Health “Guide for the Care and Use of Laboratory Animals.”

### 2.4. Transfection and Viral Transduction of ECs

HMVECs were transfected with the Bach1 siRNA (100 nmol/L) or the nontargeting negative control siRNA (100 nmol/L) using the Lipofectamine 2000 transfection reagent according to the manufacturer's instructions (Invitrogen, Carlsbad, CA). The transfection medium was replaced after six hours by EBM-2 medium and the cells were incubated for another 72 hours. Transfection efficiency was verified using reverse transcription polymerase chain reaction and Western blotting. For the viral transduction, cells were infected with the adenoviruses (Ad-Bach1 or Ad-GFP) at an MOI of 25. At 72 h after infection, cell viability was assessed. No detectable cellular toxicity was observed. Transduction was verified via GFP expression.

### 2.5. Cell Proliferation

EC proliferation was determined by bromodeoxyuridine (BrdU) incorporation into newly synthesized DNA. HMVECs were seeded in a 96-well plate in culture medium; then BrdU was added to each well and incubated with the cells for six hours. BrdU incorporation was evaluated according to the manufacturer's instructions (Calbiochem, San Diego, CA). The absorbance was measured at 450 nm using a spectrophotometer microplate reader. Each experiment was performed in triplicate, and each assay was performed three independent times.

### 2.6. Cell-Cycle Profile

HMVECs were harvested and fixed in 70% ethanol for 1 h at 4°C and then stained with a solution containing 0.05 mg/mL propidium iodide, 1 mg/mL RNase A, and 0.3% Triton X-100 in the dark for 1 h. The percentage of cells in different phases of the cell-cycle was examined by measuring the DNA content (propidium iodide intensity) with a flow cytometer (Beckman Coulter, Brea, CA), and populations of G1, S, and G2/M phase cells were determined with the ModFIT software. Each experiment was repeated three independent times.

### 2.7. Apoptosis Analysis

Cells were incubated with propidium iodide and annexin V-PE for 15 min at 37°C; then, the samples were analyzed with a flow cytometer (Beckman Coulter), and the percentage of cells that were positive for annexin V was calculated. Each experiment was performed in triplicate, and each assay was performed three times. To evaluate apoptosis in ischemic muscles, cleaved caspase 3 levels in skeletal muscle tissue homogenates were measured using Western blotting.

### 2.8. Measurement of Intracellular ROS Levels

ROS production was monitored via the dihydroethidium (DHE) assay as described previously [19]. Briefly, the cells were incubated with DHE (5 *μ*M) for 20 min in a light-protected humidified chamber at 37°C and then harvested and resuspended in PBS; then, DHE fluorescence was excited at 535 nm and monitored at 610 nm with a flow cytometer (Beckman Coulter). The ROS levels were expressed as the fold change relative to the control. MitoSOX Red was used to measure mitochondrial ROS production [20]. HMVECs were loaded with MitoSOX Red (5 *μ*M) for 15 min in a light-protected humidified chamber at 37°C, followed by washout. Cells were then incubated with Hoechst 33258 before image acquisition. Confocal images were obtained by excitation at 510 nm and measuring the emitted light at 580 nm with identical parameters for all samples. MitoSOX Red fluorescence was measured in five different areas in each sample. Immunofluorescence intensity of MitoSOX was analyzed by ImageJ software (NIH). Fluorescent levels were expressed as percent increase over the control. Each experiment was performed three times in three replicate wells.

### 2.9. Western Blot Analysis

Western blot analysis was performed as described previously [21]. Briefly, cells or tissue homogenates were lysed with RIPA buffer; then, the proteins were separated via SDS-PAGE, labeled with the corresponding antibodies and detected via chemiluminescence with a Tanon-5500 Imaging System (Tanon Science & Technology Ltd., Shanghai, China). Band intensities were quantified with the ImageJ software and normalized to the control. Each experiment was performed three times.

### 2.10. Murine Ischemic Hindlimb Model

Unilateral hindlimb ischemia was induced via femoral artery ligation as described previously [12], and then the animals were randomly assigned to treatment with intramuscular injections of Ad-Bach1 or Ad-GFP (2 × 10^8^ plaque-forming units in 40 *μ*L per mouse; *n* = 12 per experimental group). Injections were administered in the gastrocnemius muscle and the adductor muscle immediately after HLI induction.

Blood flow measurements were performed at the indicated time points with a MoorLDI2-2 laser Doppler imaging system (Moor Instruments, Devon, UK); the mice were euthanized with sodium pentobarbital (50 mg/kg i.p.) and maintained at 37°C on a heating plate to minimize temperature variation. Measurements in the ischemic limb were normalized to measurements in the nonischemic, contralateral limb.

Mice were sacrificed on day seven or 14 postsurgery and adductor muscles were harvested and snap frozen in OCT compound for cryosectioning. ROS levels were determined via DHE fluorescence. Briefly, the unfixed tissues were cut into 10 *μ*M thick sections and incubated with DHE (2 *μ*M) at 37°C for 30 min in a light-protected humidified chamber. Pictures from five random fields of each section and four sections per mouse were taken using a fluorescence microscope. Positive staining was quantified by measuring the percentage of positive staining/mm^2^ using the ImageJ software.

### 2.11. Statistical Analysis

Data are presented as the mean ± SEM. Comparisons between 2 groups were evaluated for significance with the* t*-test. Differences among groups were determined with one-way analysis of variance followed by Bonferroni post hoc test. Differences between groups were considered significant when *P* < 0.05. Analyses were performed using GRAPHPAD Prism Version 5.0 (GraphPad Software, La Jolla, CA).

## 3. Results

### 3.1. Bach1 Overexpression Promotes ROS Production and Apoptosis in the Ischemic Limbs of Mice

**Figure 1:**
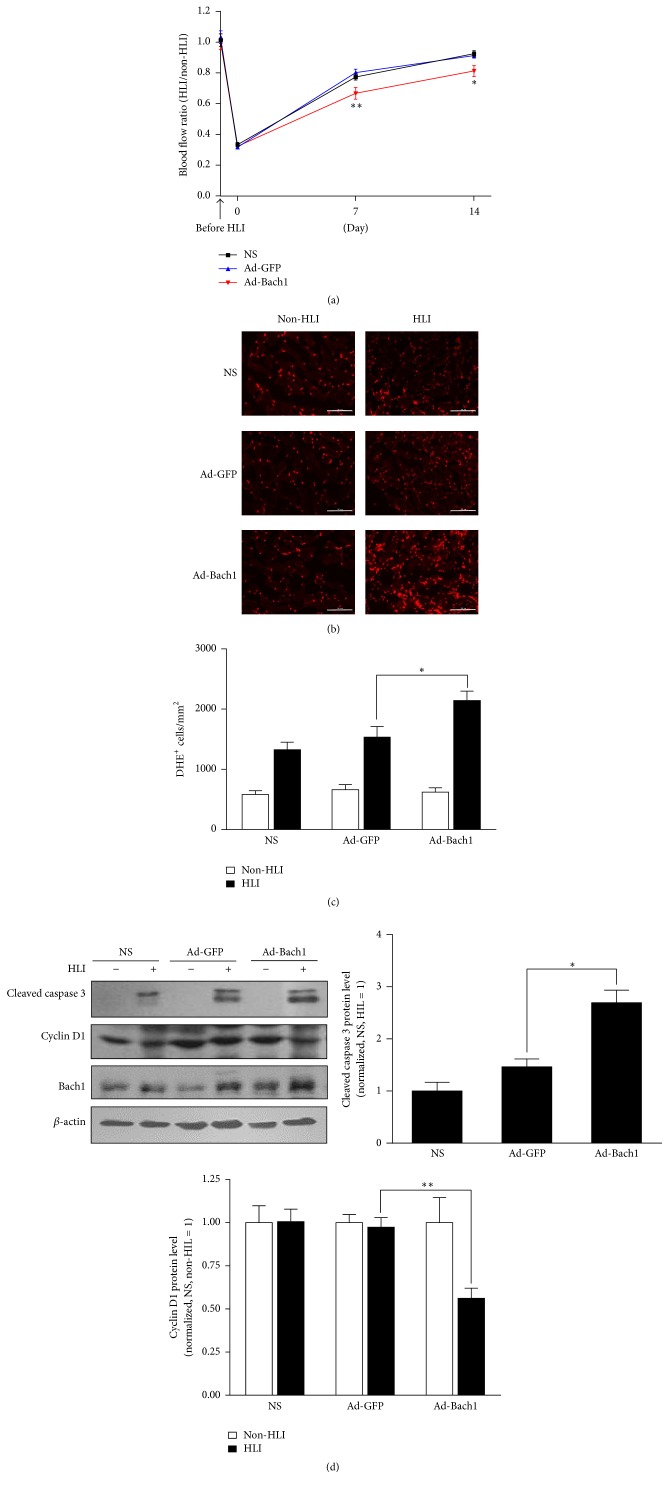
Bach1 overexpression promotes ROS production and apoptosis in the ischemic limbs of mice. Eight-week-old male C57BL/6 mice were subjected to hindlimb ischemia (HLI). GFP, Bach1 adenovirus, or saline (NS) was injected into the adductor and gastrocnemius muscles immediately after surgery. (a) Bach1 inhibited blood flow restoration after HLI. Blood flows of ischemic and nonischemic limb were measured with a laser Doppler imaging system at 0, 7, and 14 days after surgery. Ratios of blood flow from ischemic hindlimb to nonischemic hindlimb are shown (*n* = 12; ^*∗*^ *P* < 0.05, ^*∗∗*^ *P* < 0.01 versus Ad-GFP). ((b) and (c)) ROS level in nonischemic and ischemic muscle tissue on day 7 was determined by an in situ detection of superoxide with dihydroethidium (DHE) fluorescence. Bars, 100 *µ*m (b). DHE-positive cells were quantified as DHE-positive cells/mm^2^. Data from four sections of each mouse muscle tissue are shown in graphics and *n* = 6 for each group ((c), ^*∗*^ *P* < 0.05 versus Ad-GFP). (d) Seven days after HLI, cleaved caspase 3 and cyclin D1 protein levels were evaluated in HLI and non-HLI limbs via Western blot (*n* = 6; ^*∗*^ *P* < 0.05, ^*∗∗*^ *P* < 0.01 versus Ad-GFP).

Recently, we have shown that Bach1 disrupts Wnt/*β*-catenin signaling and impedes angiogenesis in a murine model of hindlimb ischemia (HLI) [12]; however, Bach1 is also known to participate in the oxidative stress response [6], cell-cycle regulation [3, 5], and apoptosis [8]. Thus, we investigated whether mechanisms involved in ROS production, cell-cycle progression, and apoptosis can be altered by upregulating Bach1 in the ischemic limbs of mice. Unilateral HLI was surgically induced by ligating the femoral artery in one hind limb, and then the injured limb was treated with intramuscular injections of adenoviruses coding for Bach1 (Ad-Bach1) or GFP (Ad-GFP) or an equal volume of normal saline (NS); two weeks later, perfusion measurements confirmed that blood flow was significantly lower in the injured limbs of Ad-Bach1 mice than in the injured limbs of mice treated with Ad-GFP or NS (Figure 1(a)). Cells with elevated ROS levels were significantly more common after treatment with Ad-Bach1 than after Ad-GFP or NS injection (Figures 1(b) and 1(c)), and Ad-Bach1 treatment was associated with significantly greater levels of cleaved caspase 3 (Figure 1(d)), which promotes apoptosis, and with lower levels of cyclin D1 (Figure 1(d)), which is required for progression through the G1 phase of the cell cycle. Thus, Bach1 overexpression appears to impede the angiogenic response to ischemic injury by increasing cellular ROS production, promoting apoptosis, and disrupting cell-cycle progression.

### 3.2. Bach1 Promotes Mitochondrial ROS Production and Apoptosis in Cultured ECs

The results from our previous investigation suggested that when Ad-Bach1 was delivered to the ischemic limbs of mice, the vectors tended to be expressed by ECs [12]. Thus, we performed a series of in vitro experiments to determine whether ROS levels, apoptosis, and cell-cycle progression can be altered in ECs by manipulating Bach1 expression. The effect of Bach1 upregulation was evaluated by comparing assessments in Ad-Bach1 infected and Ad-GFP infected HMVECs, while Bach1 downregulation was evaluated by performing experiments in HMVECs that had been transfected with Bach1 siRNA (Bach1siRNA) or a control siRNA (Con siRNA).

**Figure 2:**
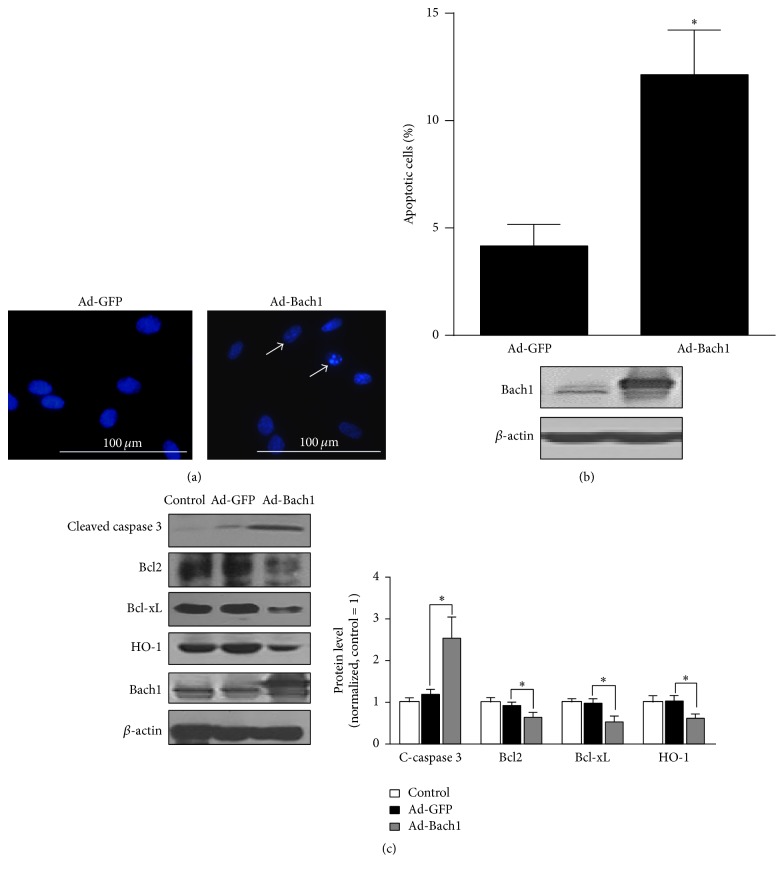
Bach1 promotes apoptosis in cultured HMVECs. (a) HMVECs were infected with the adenoviruses (Ad-GFP or Ad-Bach1); then cells were fixed at 72 hours after infection, and nuclei were visualized by Hoechst 33342. Cells with nuclear condensation are indicated by white arrows. Bars, 100 *μ*m. (b) Ad-GFP- and Ad-Bach1-infected HMVECs were seeded in 60 mm dishes, cultured for 72 hours, and then harvested and labeled with annexin V and PI. Cell apoptosis was quantified by identifying positively annexin V labeled cells via flow cytometry (*n* = 3; ^*∗*^ *P* < 0.05 versus Ad-GFP, upper panel). Bach1 protein levels were evaluated via Western blot (lower panel). (c) Cleaved caspase 3, Bcl2, Bcl-xL, HO-1, and Bach1 protein levels were determined via Western blot in HMVECs that had been infected with Ad-GFP or Ad-Bach1 (*n* = 3; ^*∗*^ *P* < 0.05 versus Ad-GFP).

**Figure 3:**
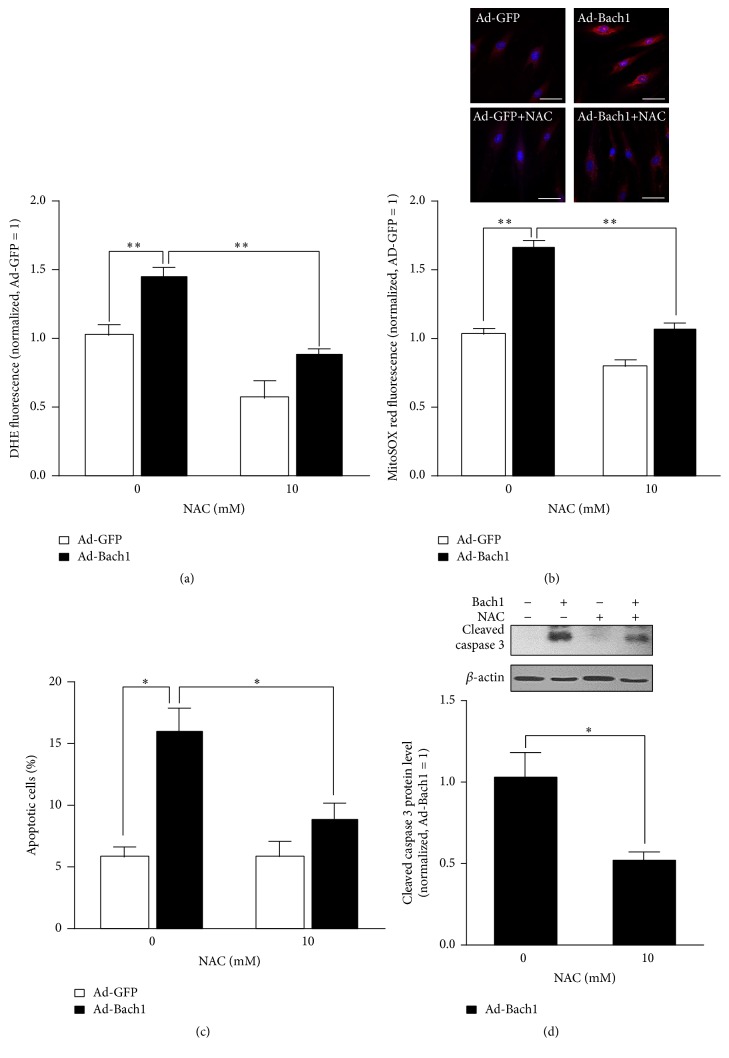
Bach1 induces apoptosis through mitochondrial ROS production. Ad-GFP- and Ad-Bach1-infected HMVECs were incubated with or without NAC (10 mM) for 48 hours, ROS production was then determined by the detection of dihydroethidium (DHE) fluorescence (a), or mitochondrial ROS production was determined by MitoSOX red fluorescence (b). Bars, 50 *μ*m. (*n* = 3; ^*∗∗*^ *P* < 0.01). Cell apoptosis (c) and cleaved caspase 3 protein levels (d) were evaluated (*n* = 3; ^*∗*^ *P* < 0.05).

Bach 1 overexpression appeared to promote apoptotic nuclear condensation (Figure 2(a)) and cell apoptosis (Figure 2(b)), and cleaved caspase 3 levels (Figure 2(c)) and intracellular ROS levels (Figure 3(a)) were significantly higher in populations of Ad-Bach1 HMVECs than in Ad-GFP HMVECs. MitoSOX Red, a redox fluorophore detecting selectively mitochondrial superoxide, was used to evaluate mitochondrial ROS generation in HMVECs. The fluorescence intensity of MitoSOX Red was significantly higher in Ad-Bach1 HMVECs than that in Ad-GFP HMVECs (Figure 3(b)), indicating that Bach1 increases mitochondrial ROS levels. The higher levels of Bach1 expression was also associated with declines in expression of the apoptosis inhibitors Bcl2, Bcl-xL, and heme oxygenase 1 (HO-1) (Figure 2(c)). However, when Ad-Bach1 HMVECs were cultured with the ROS scavenger N-acetyl-L-cysteine (NAC; 10 mM), mitochondrial ROS levels (Figure 3(b)), cell apoptosis (Figure 3(c)), and cleaved caspase 3 levels (Figure 3(d)) declined significantly.

**Figure 4:**
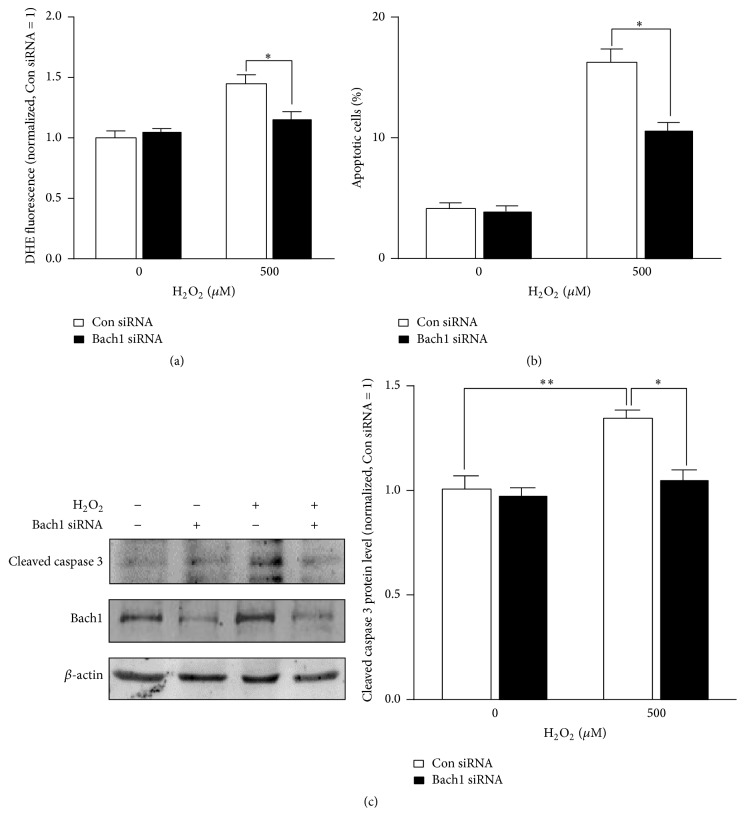
Bach1 downregulation attenuates H_2_O_2_-induced cell apoptosis. HMVECs were transfected with Con siRNA or Bach1 siRNA for 72 hours and then treated with or without H_2_O_2_ (500 *μ*M) for 12 hours; the levels of ROS (a) cell apoptosis (b) and the protein levels of cleaved caspase 3 and Bach1 (c) were evaluated (*n* = 3; ^*∗*^ *P* < 0.05, ^*∗∗*^ *P* < 0.01).

Measurements in Bach1siRNA- and Con siRNA-transfected cells were generally similar under normal culture conditions, but when oxidative stress was induced by culturing the cells with 500 *μ*M hydrogen peroxide, Bach1 silencing was associated with significant declines in ROS levels (Figure 4(a)), cell apoptosis (Figure 4(b)), and cleaved caspase 3 levels (Figure 4(c)). Collectively, these observations suggest that Bach1 activates ROS-dependent apoptotic signaling pathways in ECs.

### 3.3. Bach1 Disrupts Cell-Cycle Progression and Limits Proliferation in Cultured ECs

**Figure 5:**
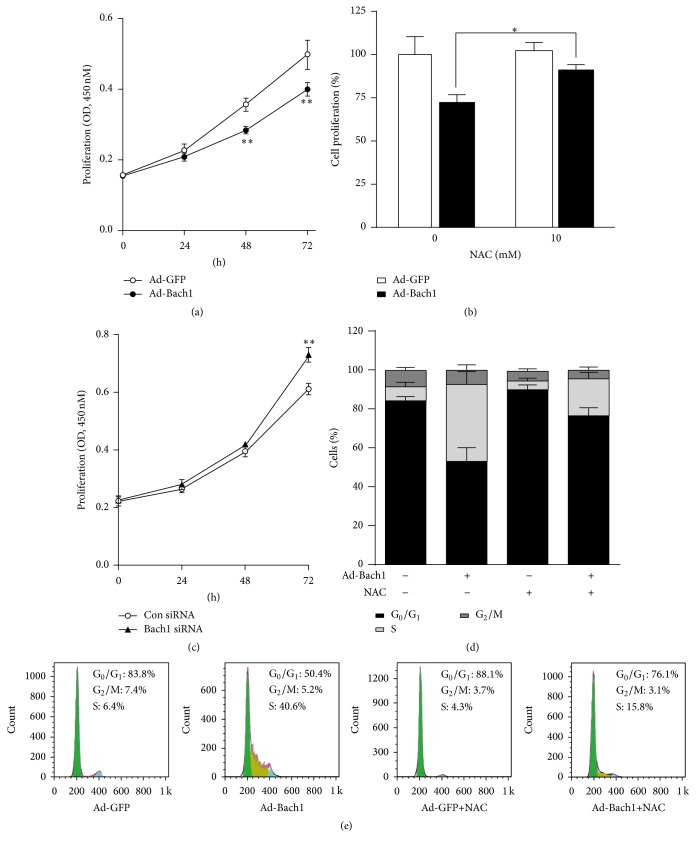
Bach1 disrupts cell-cycle progression and inhibits cell proliferation through ROS production. Cell proliferation was evaluated in (a) HMVECs that had been infected with Ad-GFP or Ad-Bach1, in (b) Ad-GFP- and Ad-Bach1-infected HMVECs treated with or without NAC (10 mM) for 48 hours, and in (c) HMVECs that had been transfected with Bach1siRNA or Con siRNA. Cells were cultured in 96-well plates, and Brdu incorporation assessments were performed via optical density measurements (450 nm wavelength) at the indicated time points (*n* = 3; ^*∗*^ *P* < 0.05, ^*∗∗*^ *P* < 0.01). ((d) and (e)) HMVECs were infected with Ad-GFP or Ad-Bach1 and then incubated with or without NAC (10 mM) for 48 hours; then cells were harvested. The cell-cycle analysis was conducted using flow cytometry and quantified (*n* = 3).

**Figure 6:**
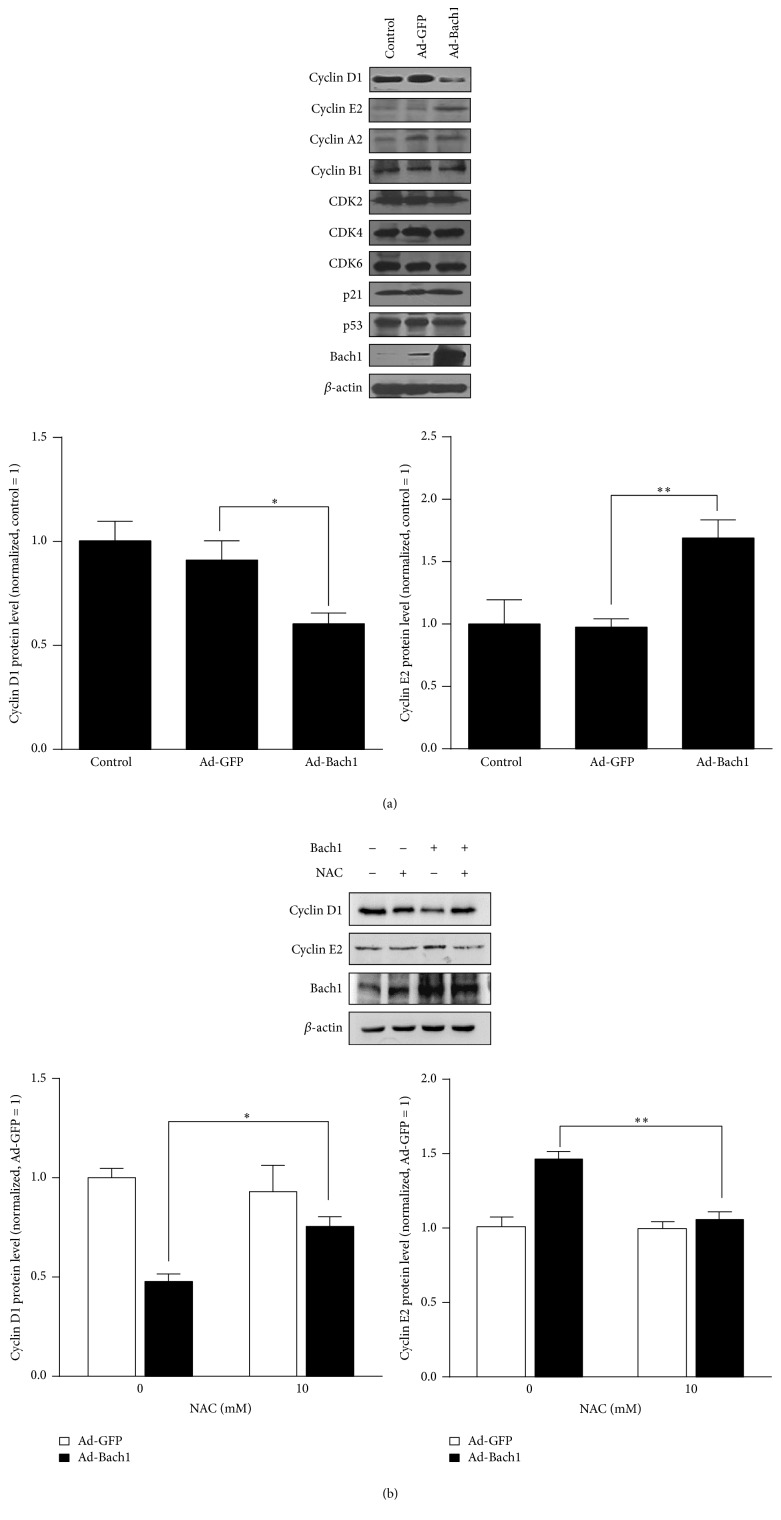
Bach1 regulates the expression of cell-cycle regulatory proteins through ROS production. (a) Western blot analysis of cell-cycle regulatory proteins (cyclin D1, cyclin E2, cyclin A2, cyclin B1, CDK2, CDK4, CDK6, p21, and p53) in Ad-GFP- and Ad-Bach1-infected HMVECs (*n* = 3; ^*∗*^ *P* < 0.05, ^*∗∗*^ *P* < 0.01). (b) HMVECs were infected with Ad-GFP or Ad-Bach1 and then incubated with or without NAC (10 mM) for 48 hours; then cells were harvested. Cyclin D1, cyclin E2, and Bach1 protein levels were evaluated (*n* = 3; ^*∗*^ *P* < 0.05, ^*∗∗*^ *P* < 0.01).

Bach1 overexpression was associated with a significant decline in cell proliferation (Figure 5(a)), and this decline was partially abolished by culturing the cells with NAC (Figure 5(b)). However, cell proliferation was significantly greater in Bach1siRNA than in Con siRNA HMVECs (Figure 5(c)), even though apoptosis rates for the two cell populations were similar (Figure 4(b)). Bach1 overexpression also caused a decrease of cells in the G0/G1 phase and an increase of cells in the S phase, and this S-phase cell-cycle arrest was partially blocked by concurrent treatment with NAC (Figures 5(d) and 5(e)). Furthermore, cyclin D1 levels were significantly reduced in Ad-Bach1 HMVECs but increased significantly when the cells were cultured with NAC (Figures 6(a) and 6(b)). Cyclin E2 levels were significantly greater in Ad-Bach1 HMVECs but decreased markedly when the cells were treated with NAC (Figures 6(a) and 6(b)). Thus, the decline in cell proliferation associated with Bach1 overexpression likely evolved, at least in part, from ROS-induced changes in cell-cycle progression.

## 4. Discussion

**Figure 7:**
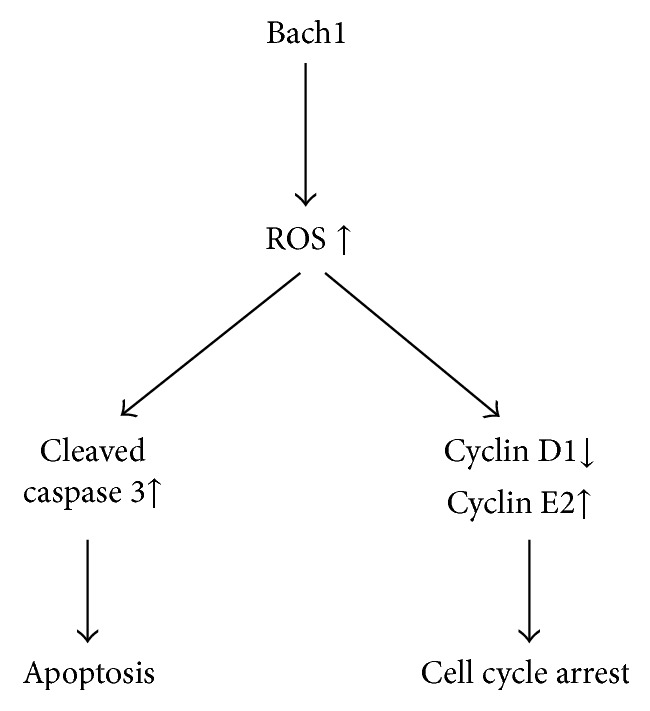
Mechanisms for the Bach1-induced cell-cycle arrest and apoptosis in ECs. Bach1 induces caspase 3-dependent apoptosis by ROS production in ECs; Bach1 also regulates the expression of cell-cycle regulatory proteins (cyclin D1 and cyclin E2) and promotes cell-cycle arrest through ROS generation.

Bach1 is known to regulate the expression of genes involved in the oxidative stress response and cell-cycle progression [1–5], and we have recently shown that Bach1 impairs proliferation and promotes apoptosis in cultured ECs [12]. Thus, we manipulated the level of Bach1 expression in the limbs of mice with HLI and in cultured HMVECs to determine whether the proapoptotic activity of Bach1 is mediated by ROS production. Our results indicated that Bach1 upregulation impaired the blood flow recovery from hindlimb ischemia and this effect was accompanied both by increases in ROS and cleaved caspase 3 levels and by declines in the expression of cyclin D1 in the injured tissues. Bach1 also promoted mitochondrial ROS production and the levels of cleaved caspase 3 in cultured HMVECs and disrupted cell-cycle progression, and these effects were largely abolished when the cells were cultured with the ROS scavenger NAC. Thus, Bach1 appears to impede cell-cycle progression and induce apoptosis in ECs through increases in mitochondria ROS production (Figure 7).

Bach1 target involved in the oxidative stress response includes heme oxygenase-1 (HO-1). We found that HO-1 was downregulated in Bach1-overexpressing cells, which plays a crucial role in protection from oxidative stress and apoptosis [22, 23], and HO-1 catabolizes heme into ferrous iron, carbon monoxide, and biliverdin, which have antioxidant and antiapoptotic properties in vivo [24]. Another Bach1 target involved in the oxidative stress response is the glutamate-cysteine ligase modifier subunit (GCLM) [25], which increases synthesis of the antioxidant glutathione and was upregulated after Bach1 knockdown [3]. Thus, Bach1 likely increases ROS generation by inhibiting antioxidants, at least, including HO-1. Most ROS are generated in cells by the mitochondrial respiratory chain. Mitochondrial ROS production is modulated largely by the rate of electron flow through respiratory chain complexes [26]. We showed that mitochondria were the major source of Bach1-induced ROS generation in HMVECs. ROS can also be generated by the activation of various enzymes, including mitochondrial oxidases, the NADPH oxidases, NO synthases, and xanthine oxidase [27]. The specific mechanisms linking Bach1 and ROS production have yet to be identified in ECs.

We have recently demonstrated that Bach1 upregulation can impair angiogenesis in murine models of peripheral ischemic injury [12]. In the present study, we found that Bach1 overexpression enhanced ROS production and induced apoptosis in ischemic mouse hindlimbs. High levels of ROS have been shown to increase apoptosis and impair neovascularization in the ischemic limbs of mice [16, 17]. Thus, Bach1 likely inhibits angiogenesis at least partially by increasing ROS generation and apoptosis.

Apoptosis can be induced either through an extrinsic pathway, which is triggered by the binding of apoptosis-inducing ligands to cell surface receptors, or through an intrinsic pathway, which is regulated by the balance between proapoptotic and antiapoptotic Bcl2 proteins at the mitochondria [28–30]. In the present study, both Bcl2 and Bcl-xL levels declined in response to Bach1 upregulation in HMVECs. ROS have been shown to induce mitochondrial injury by reducing mitochondrial membrane potential and decreasing the expression ratio of Bcl2/Bax [31, 32]. Thus, the suppression of Bcl2 and Bcl-xL by Bach1 overexpression may be due to the increase of ROS production in cells. In addition, we also found that Bach1 triggered caspase 3 activation, and caspase 3 can be cleaved and activated by components of both pathways [33, 34]. Thus, the relationship between Bach1 levels and ROS production could contribute to both extrinsic and intrinsic apoptotic signaling.

Cyclin D1 and E2 were not among the direct Bach1-targeted cell-cycle-related proteins identified in HEK293 cells [3]. However, we have previously shown that Bach1 suppresses the Wnt/*β*-catenin pathway [12], which is known to regulate cyclin D1, and previous studies have linked increases in cyclin E expression with S-phase arrest [35]. Thus, Bach1 appears to inhibit cell-cycle progression and proliferation in ECs through a network of at least three overlapping mechanisms: (1) declines in Wnt-mediated cyclin D1 expression, (2) increases in ROS-mediated cyclin D1 repression, and (3) upregulation of cyclin-E2-induced S-phase arrest.

In conclusion, our results indicate that Bach1 inhibits cell proliferation and induces cell-cycle arrest and apoptosis by increasing mitochondria derived ROS production and, consequently, that functional downregulation of Bach1 may be a promising treatment target for the treatment of vascular diseases.
